# Study on the Experience of Public Health System Construction in China's COVID-19 Prevention

**DOI:** 10.3389/fpubh.2021.610824

**Published:** 2021-04-23

**Authors:** Pengfei Zhang

**Affiliations:** School of Labor and Human Resources, Renmin University of China, Beijing, China

**Keywords:** public health system construction, COVID-19, prevention, experience, China

## Abstract

**Background:** China's experience in the process of COVID-19 prevention provides a reference for other countries in the world. This article studied the experience of public health system construction in China's COVID-19 prevention.

**Methods:** Based on literature review and theoretical analysis, this paper constructs a theoretical framework of national public health system construction in health crisis. Based on this theoretical framework, combined with the policies and measures formulated by the Chinese government in the process of COVID-19 prevention, this article evaluate the advantages and deficiencies of China's public health system construction in response to COVID-19.

**Results:** The Chinese government ensured the adequate supply of health resources, improved people's ability to pay medical expenses, and adopted advanced public health propaganda methods based on the Internet to help people grasp the basic information and development trend of COVID-19 in the process of COVID-19 prevention. At the same time, the utilization efficiency of health resources was low in China, people's ability to pay for medical expenses was unequal, and the disclosure of virus information in the early stage of the outbreak of COVID-19 is not timely.

**Conclusions:** Other countries can learn from the advantages of China's public health system construction and avoid China's deficiencies in the process of public health system construction, which will help them improve the efficiency of COVID-19 prevention.

## Introduction

COVID-19 appeared in Wuhan, China in December 2019. Just 1 month later, COVID-19 appeared in other countries in the world. Compared with the previously discovered coronaviruses, COVID-19 has outstanding characteristics, which brings new challenges to the public health system construction of various countries in the world. First of all, COVID-19 is highly contagious, which makes it rapidly spread to most countries in the world (1). When most countries find signs of COVID-19, there is often a large area of COVID-19 infection, which makes the public health system construction in most countries difficult to respond to the development of COVID-19 in time. Second, the strong infectivity of COVID-19 causes many people to be infected, which leads to the shortage of health resources for the treatment of COVID-19 (2, 3). Especially for underdeveloped countries, unsound medical technology prevents the infected people from getting timely and effective treatment, which increases the risk of death. Third, COVID-19 has strong variability, and the infectivity and mortality of the mutated virus may be greatly improved, which may cause the process of public health system construction in response to the COVID-19 is long (4, 5). COVID-19 has brought serious harm to world economic development and social stability. For example, in the face of the impact of COVID-19, some countries have to restrict population mobility and shut down some living facilities and work departments, which in turn affect people's lives and incomes, and even exacerbates poverty (6, 7).

Public health system construction plays an important role in the process of COVID-19 prevention. Especially for developing countries and underdeveloped countries, public health system construction in response to COVID-19 can help them reduce the infection rate of COVID-19 and improve the prevention efficiency of COVID-19 (8, 9). As the most populous country in the world, China's experience in the process of COVID-19 prevention provides a reference for other countries in the world. China's public health system construction is a huge project. Especially in the face of the sudden COVID-19, it is a huge challenge to protect the medical demand of more than 1.4 billion people. Moreover, the scale of population mobility in China is huge. Facing the strong infectivity of COVID-19, the huge scale of population mobility undoubtedly aggravates the risk of cross infection of the virus. In addition, 1.4 billion people need a lot of health resources in the process of COVID-19 prevention, and ensuring people's access to medical services is also a major problem in public health system construction in China. In fact, with such a large population, China has controlled the large-scale spread of COVID-19 to a certain extent through public health system construction in the process of prevention. Moreover, in the process of COVID-19 prevention, China has ensured that people infected with COVID-19 can receive timely treatment through public health system construction. In addition, there are also some problems in the process of public health system construction in China. These problems also have certain reference value for other countries, which can help other countries avoid repeating the same mistakes in the process of public health system construction in response to COVID-19. This article studied the experience of public health system construction in China's COVID-19 prevention.

## Methods

This article adopts a qualitative design. Based on literature review and theoretical analysis, this paper constructs a theoretical framework of national public health system construction in health crisis. Based on this theoretical framework, combined with the policies and measures formulated by the Chinese government in the process of COVID-19 prevention, this article evaluate the advantages and deficiencies of China's public health system construction in response to COVID-19.

For any country, the purpose of public health system construction is to protect people's health. The emergence of health crises brings both physical and psychological effects to people. Different from ordinary diseases, diseases that trigger health crises often have higher infectious and mortality rates (10, 11). Once people are infected by a disease that triggers a health crisis, their demand for health resources is often several times higher than usual (12, 13). In a peaceful period without health crises, the supply of health resources can basically maintain people's normal demand. However, in the period of health crisis, health resources are often in short supply. There are two reasons for the shortage of health resources during the health crisis. On the one hand, people's demand for health resources rose sharply during the health crisis, which broke the balance of supply and demand of health resources in the original peaceful period without health crises. For example, with the spread of health crisis, the number of infected people is gradually increasing, and the corresponding demand for health resources is also gradually rising (14). Moreover, the psychological panic caused by the health crisis will also lead people to ease their worries by increasing the reserve of health resources, which will also cause an unreasonable increase in the demand for health resources (15). On the other hand, in order to control the spread of the health crisis, the government is forced to formulate measures to curb population mobility, and a large part of which involves the closure of the production sectors. The closure of production sectors will hinder the production of health resources and lead to insufficient supply (16, 17). Therefore, a country's public health system construction must ensure adequate health resources during the COVID-19 prevention.

The health crisis will also cause a decline in people's ability to bear medical expenses (18, 19). The decline in the ability to pay for medical expenses makes it difficult for people to maintain their health. Especially for the poor, their ability to pay for medical expenses in peaceful period without health crises is inherently low. The occurrence of a health crisis makes it difficult for them to afford the cost of maintaining health, and they are ultimately forced to give up treatment (20, 21). This phenomenon is particularly common in developing countries and underdeveloped countries. There are two reasons for the decline of people's ability to pay medical expenses during health crisis. First, health resources become scarcer during the health crisis, leading to an increase in the price of health resources. During the health crisis, people's demand for health resources increased, breaking the balance of supply and demand of health resources in peaceful period without the health crisis. The demand for health resources was greater than the supply during the health crisis, leading to an increase in the price of health resources (22). Second, during the health crisis, the country's economic development will be affected, leading to a decline in people's income. For example, some countries are forced to adopt the method of closing work sectors to control the spread of the health crisis, which will inevitably affect people's income (23, 24). It can be seen that during the health crisis, the rise in the price of health resources and the decline in people's income lead to a decline in people's ability to pay for medical expenses. Therefore, a country's public health system construction must protect people's ability to pay for medical expenses during the COVID-19 prevention.

The harm of health crisis to people's psychology also deserves the attention of the public health system. The psychological harm of health crisis to people is invisible, and once the psychological harm is formed, it is often difficult to cure completely. Therefore, the psychological harm caused by health crisis is more serious than the physical harm. The ways of health crisis to people's psychological harm mainly come from two aspects. On the one hand, the psychological harm comes from people's ignorance of the virus that causes health crisis (25, 26). Because health crisis is sudden and the virus causing health crisis is highly infectious, once the health crisis is disclosed to people, the virus usually has spread over a large area. From the health crisis being disclosed to people fully grasping the virus information, there is a vacuum period of people's cognition of the virus that causes the health crisis. This vacuum period will increase people's panic and anxiety, and then cause psychological problems. This is because the occurrence of health crisis increases the risk of people's living environment. In the vacuum period, if people do not grasp information about the virus, they will not be able to judge whether their environment is at risk, which will obviously increase people's unnecessary anxiety and tension. On the other hand, the psychological harm comes from unknown of the development trend of the health crisis (27, 28). Once a health crisis occurs in a country, it often takes a long time for the country to prevent it. Especially for the health crisis caused by the virus with strong variability, its development trend is often difficult to predict. The unpredictability of health crisis disturbs people's long-term planning for their future life and work, triggers people's worries, and easily leads to psychological harm. Therefore, a country's public health system construction must focus on alleviating the psychological harm of health crisis to people during the COVID-19 prevention.

Based on the above three dimensions of the public health system construction in the process of COVID-19 prevention, we evaluated the advantages and deficiencies of the China's experience combined with the policies and measures formulated by the Chinese government.

## Results

### Adequate Supply of Health Resources and Inefficient Utilization of Health Resources

For China with 1.4 billion people, the amount of health resources consumed every day is huge. Especially after the outbreak of COVID-19, people's demand for health resources is several times higher than before. In the process of China's public health system construction, the Chinese government has fully considered the impact of possible health crises on the supply of health resources. The Chinese government has established a reserve system of health resources, which meets people's demand for health resources during the COVID-19 prevention. First of all, under the reserve system of health resources, the Chinese government has built a batch of health resources for preventing health crises through fiscal expenditure, involving hospitals, doctors, and medical equipment. After the emergence of COVID-19, these reserved health resources were quickly applied to the prevention work (29). Second, unlike other countries, the public health power of the Chinese government is relatively high, which can make the Chinese government spend more fiscal expenditure and human resources on the construction of temporary health resources in the process of COVID-19 prevention. For example, during the COVID-19 prevention, the Chinese government had spent a lot of fiscal expenditure and human resources to build temporary hospitals in the short term to ensure timely treatment of infected people (30). The technical level of doctors and the improvement level of medical facilities in these temporary hospitals are the same as those in normal hospitals. Furthermore, the higher public health power makes it easier for the Chinese government to regulate the regional mobility of health resources. During the spread of COVID-19, all regions in China can be divided into high-risk regions and low-risk regions. People in low-risk regions have relatively low demand for health resources, while people in high-risk regions have relatively high demand for health resources. Therefore, the remaining health resources in low-risk regions can be allocated to high-risk regions. The Chinese government ensures the adequate supply of health resources in high-risk regions through the allocation of health resources from low-risk regions to high-risk regions (31).

At the same time, the inefficient utilization of health resources is one of the important deficiencies of China's public health system construction during the COVID-19 prevention. COVID-19 caused people's panic, forcing people to start hoarding a lot of health resources. The amount of health resources that people actually hoard is far greater than their actual demand, resulting in the waste of health resources and reducing the utilization efficiency of health resources (32). Moreover, people's hoarding of health resources triggered by the panic makes those who really need health resources may not be able to meet the demand of health resources in time. In order to protect the demand of people who really need health resources, the Chinese government is forced to build more health resources than the actual demand, which causes the waste of financial expenditure and human resources to a certain extent (33). In addition, although the Chinese government allocates resources from low-risk regions to high-risk regions to meet the health resources demand of people in high-risk regions, rural regions with low levels of economic development still face the shortage of health resources (34). The technical level of doctors and the improvement level of medical facilities in rural regions are relatively low. Faced with the threat of COVID-19, it is difficult to effectively protect the health of residents in rural regions. Compared with rural regions, urban regions in China are rich in health resources, and even have a lot of surpluses of health resources. These surpluses of health resources are not allocated to rural regions in time, resulting in low utilization efficiency of health resources.

### The Improvement of the Ability to Pay Medical Expenses and the Inequality of the Ability to Pay Medical Expenses

During the COVID-19 prevention, in order to improve people's ability to pay for medical expenses, the Chinese government strengthened the connection between medical insurance system and COVID-19 treatment in the process of the public health system construction. First, for people who are infected with COVID-19, they can improve their ability to pay for medical expenses through the medical insurance system. Specifically, part of the medical expenses of people infected with COVID-19 are paid by the medical insurance fund in the process of treatment, and people only pay part of the medical expenses (35). Second, the Chinese government implements medical assistance measures for the poor. Medical assistance measures are part of the medical insurance system and mainly for the poor. When the poor are infected with COVID-19, because they can't afford the high expenses of treatment, the Chinese government helps them to pay most of the medical expenses through medical assistance measures (36). The support funds for medical assistance measures come from fiscal expenditure. In addition, the improvement of medical insurance on the ability to pay medical expenses of people infected with COVID-19 is not only reflects in the treatment, but also involves the reduction of medical insurance contributions. In China, the support fund of medical insurance system comes from people's contributions. During the COVID-19 prevention, the Chinese government has reduced people's medical insurance contributions, which can reduce people's economic pressure to a certain extent and improve people's ability to pay for medical expenses (37).

For the prevention of COVID-19, although the medical insurance system improved people's ability to pay for medical expenses in the process of China's public health system construction, a new deficiency also emerged. There are differences in the improvement of people's ability to pay for medical expenses by the medical insurance system, which causes inequality. China's medical insurance system is divided into different types of projects according to different groups of people (38). For employees in urban regions, they participate in the Basic Medical Insurance for Urban Employees (BMIUE). For non-employees in urban regions, they participate in the Basic Medical Insurance for Urban Residents (BMIUR). For the population in rural regions, they participate in the New Rural Cooperative Medical Insurance (NRCMI). These three types of projects pay different medical expenses for people infected with COVID-19. Among them, the BMIUE pays the highest medical expenses for people infected with COVID-19, and it has the best effect in improving people's ability to pay for medical expenses. While the NRCMI pays the lowest medical expenses for people infected with COVID-19, and its ability to improve people's ability to pay for medical expenses is the worse than the BMIUE and the BMIUR (39). Therefore, for people participating in the NRCMI, when they are infected by COVID-19, the difference of medical insurance types leads to their low ability to pay for medical expenses. Therefore, the inequality of the ability to pay medical expenses is caused by the imperfect design of the medical insurance system. This problem can only be solved by the reform of medical insurance system, not by people themselves, because the medical insurance types are divided according to urban regions and rural regions. In addition, the difference in the level of economic development increased the difference in the ability to pay for medical expenses between rural residents and urban residents. In the face of the COVID-19, rural residents and urban residents bear the same economic pressure on medical expenses. However, rural residents' low ability to pay for medical expenses makes them difficult to maintain health, and even forced to give up treatment.

### Advanced Public Health Propaganda Methods and Untimely Information Disclosure

China is a country that pays special attention to information propaganda. As early as in the period of planned economy system, China explored various ways to propagate information to people. The reason why China pays attention to information propaganda is that China has a large population. In order to ensure that the central government's information is disseminated to most people, various propaganda methods must be explored. In the process of China's public health system construction, advanced propaganda methods were also fully used to propagate COVID-19 information. On the one hand, during the COVID-19 prevention, the Chinese government disseminated the development information of COVID-19 to people through the Internet based on information technology (40). Compared with traditional information propaganda methods, COVID-19 information propaganda methods based on the Internet can greatly reduce unnecessary cross-infection, which is conducive to reducing the spread of COVID-19. On the other hand, in the process of deploying human resources and disclosing the specific preventive measures of COVID-19, the Chinese government also adopted information propaganda methods based on the Internet. The Chinese government formulated a series of COVID-19 prevention measures through Internet conferences and made the information public through the Internet, which saved a lot of time and improved the efficiency of COVID-19 prevention work (41). In addition, during the inspection of the population infected by COVID-19, medical institutions also spread basic information and detailed preventive measures of COVID-19 to the society through Internet, which enables people to regulate their own life behaviors and reduces the risk of being infected with COVID-19 (40). Through advanced public health propaganda methods based on the Internet, people can grasp the development trend of COVID-19, detailed basic information of COVID-19 and preventive measures, so as to reduce people's unknowns and worries about COVID-19, which is conducive to alleviating the psychological harm caused by COVID-19 to people.

Although China has advanced public health propaganda methods, the disclosure of the virus information in the early stage of the COVID-19 outbreak was not timely. The early stage of the COVID-19 outbreak is a key stage to control the spread of the virus. However, at this critical stage, the Chinese government did not take timely measures, and did not disclose COVID-19 information to the society in time. This deficiency of the public health system is fatal. Due to this deficiency, COVID-19 spread rapidly and eventually spread to all provinces in China. At the same time, people did not take any preventive measures against the spread of COVID-19 because they did not receive timely information disclosure. Untimely information disclosure caused the Chinese government's follow-up preventive measures to become passive, and more and more people were infected by COVID-19, which had a serious impact on people's health (42). Moreover, untimely information disclosure increased people's panic about COVID-19 and brought psychological harm to people. Especially for employees, untimely information disclosure reduced the time for them to adjust their work plans, causing some employees to lose their work income and face the risk of falling into poverty (43). Furthermore, untimely information disclosure reduces people's trust in the Chinese government and public health system, which brings difficulties to the implementation of follow-up COVID-19 prevention work. For example, in the process of COVID-19 prevention, untimely information disclosure made some people hold a negative attitude toward the preventive measures formulated by the Chinese government, which hindered people from cooperating with the Chinese government.

## Discussion

Based on the theoretical framework of national public health system construction in health crisis and combined with the policies and measures formulated by the Chinese government in the process of COVID-19 prevention, this article studied the advantages and deficiencies of China's public health system construction in response to COVID-19. The study found that the Chinese government ensured the adequate supply of health resources, improved people's ability to pay medical expenses, and adopted advanced public health propaganda methods based on the Internet to help people grasp the basic information and development trend of COVID-19 in the process of COVID-19 prevention. These advantages of China's public health system construction meet people's medical demand to a certain extent, improve people's access to medical services, and increase people's awareness of COVID-19 prevention. However, there were still deficiencies in the process of COVID-19 prevention. The study found that the utilization efficiency of health resources was low in China, people's ability to pay for medical expenses was unequal, and the disclosure of virus information in the early stage of the outbreak of COVID-19 is not timely. These deficiencies brought serious challenges to China's COVID-19 prevention. The utilization efficiency of health resources caused a serious waste of health resources and made the Chinese government forced to invest a lot of financial funds for the construction of health resources, resulting in a waste of financial funds. People's unequal ability to pay for medical expenses originated from the defect of medical insurance system, which exacerbated the opposition of different groups. Especially for urban residents and rural residents, they participated in two different projects in the process of COVID-19 prevention, which made it difficult for rural residents to effectively protect their health. Untimely information disclosure in the early stage of the outbreak of COVID-19 was fatal for both the COVID-19 prevention and the social order. Untimely information disclosure accelerated the spread of COVID-19, which led to the development of COVID-19 from some regions to all provinces in China.

In response to COVID-19, China's public health construction should focus on improving the utilization efficiency of health resources. On the one hand, the allocation of healthy resources should depend on the population size of the region, rather than the level of economic development of the region. Health is the basic demand of people, and the amount of health resources should be related to the population size, not to the level of economic development. Especially for rural areas, the Chinese government should promote the mobility of health resources from cities to rural areas, and improve the infrastructure health facilities in rural areas, which is conducive to improving the health level of rural residents and improving the utilization efficiency of health resources. On the other hand, the allocation of health resources should depend on the development trend of COVID-19. People in high-risk areas have high demand for health resources, while people in low-risk areas have low demand for health resources. The Chinese government should promote the mobility of health resources from low-risk areas to high-risk areas. In high-risk areas, it is necessary for the Chinese government to correctly guide people's demand of health resources, and promote people to use health resources rationally, instead of excessively hoarding health resources.

The Chinese government should reform the medical insurance system to reduce the difference between urban residents and rural residents in their ability to pay for medical expenses, which is conducive to enhancing the rural residents' access to medical services in the process of prevention of COVID-19. Because the level of economic development in rural areas is lower than that in urban areas, and the income level of rural residents is significantly lower than that of urban residents, the ability of rural residents to pay for medical expenses is lower than that of urban residents. However, this gap has not been solved through the medical insurance system, but has been further expanded by the medical insurance system. Whether people are urban residents or rural residents, the medical expenses for treatment after being infected by COVID-19 will not vary with their status. Therefore, only by reforming the medical insurance system and strengthening the protection of rural residents' medical demand, can the Chinese government effectively solve the problem of rural residents' low ability to pay for medical expenses.

Moreover, the Chinese government should ensure that COVID-19 information can be disclosed to the public in time. Virus information cannot be concealed, and it will eventually be discovered by the public in a spreading way. In the early stage of the COVID-19 outbreak, untimely information disclosure not only exposed people to the virus, but also aggravated the spread of the virus. The Chinese government should learn from this deficiency. In addition, the Chinese government should improve the efficiency of virus detection, so that the virus can be detected in time at the early stage of virus development. High efficiency of virus detection can help the government formulate timely preventive measures, which is conducive to significantly controlling the spread of the virus.

## Conclusion

Through the study on the experience of public health system construction in China's COVID-19 prevention, this article analyzed the advantages and deficiencies of China's public health system construction in response to COVID-19. This study has important reference value for other countries in the world by analyzing the COVID-19 prevention experience of China. Other countries should ensure people's health demand through the adequate supply of health resources, ensure people's access to medical services by improving people's ability to pay for medical expenses, and raise people's awareness of COVID-19 by adopting advanced public health propaganda methods. At the same time, they should avoid the deficiencies in the process of China's public health system construction. During the COVID-19 prevention, they should avoid the phenomenon of inefficient utilization of health resources, promote equality in people's ability to pay for medical expenses, and disclose the development trend of COVID-19 to people in time, which will help them improve the efficiency of COVID-19 prevention.

## Data Availability Statement

The original contributions presented in the study are included in the article/Supplementary Materials, further inquiries can be directed to the corresponding author.

## Ethics Statement

The studies involving human participants were reviewed and approved by Committee at Renmin University of China. The patients/participants provided their written informed consent to participate in this study.

## Author Contributions

PZ designs the study and drafted the manuscript.

## Conflict of Interest

The author declares that the research was conducted in the absence of any commercial or financial relationships that could be construed as a potential conflict of interest.

## References

[1] Hatefi S Smith F Abou-El-Hossein K Alizargar J. COVID-19 in South Africa: lockdown strategy and its effects on public health and other contagious diseases. Public Health. (2020) 185:159–60. 10.1016/j.puhe.2020.06.033 32629201 PMC7303625

[2] Diptyanusa A Zablon KN. Addressing budget reduction and reallocation on health-related resources during COVID-19 pandemic in malaria-endemic countries. Malar J. (2020) 19:411. 10.1186/s12936-020-03488-y 33198747 PMC7668022

[3] Apriceno M Lytle A Monahan C Macdonald J Levy SR. Prioritizing health care and employment resources during COVID-19: roles of benevolent and hostile ageism. Gerontologist. (2021) 61:98–102. 10.1093/geront/gnaa165 33119089 PMC7665451

[4] Hadied MO Patel PY Cormier P Poyiadji N Salman M Klochko C. Interobserver and intraobserver variability in the CT assessment of COVID-19 based on RSNA consensus classification categories. Acad Radiol. (2020) 27:1499–506. 10.1016/j.acra.2020.08.038 32948442 PMC7492048

[5] Benetti E Tita R Spiga O Ciolfi A Birolo G Bruselles A. ACE2 gene variants may underlie interindividual variability and susceptibility to COVID-19 in the Italian population. Eur J Hum Genet. (2020) 28:1602–14. 10.1038/s41431-020-0691-z 32681121 PMC7366459

[6] Pereira M Oliveira AM. Poverty and food insecurity may increase as the threat of COVID-19 spreads. Public Health Nutr. (2020) 23:3236–40. 10.1017/S1368980020003493 32895072 PMC7520649

[7] Sliwa K Yacoub M. Catalysing the response to NCDI Poverty at a time of COVID-19. Lancet. (2020) 396:941–3. 10.1016/S0140-6736(20)31911-5 32941824 PMC7489991

[8] Dixon BE Caine VA Halverson PK. Deficient response to COVID-19 makes the case for evolving the public health system. Am J Prev Med. (2020) 59:887–91. 10.1016/j.amepre.2020.07.024 32978011 PMC7448961

[9] Carter P Anderson M Mossialos E. Health system, public health, and economic implications of managing COVID-19 from a cardiovascular perspective. Eur Heart J. (2020) 41:2516–8. 10.1093/eurheartj/ehaa342 32320040 PMC7188109

[10] Filippidis FT Gerovasili V Man WD Quint JK. Trends in mortality from respiratory system diseases in Greece during the financial crisis. Eur Respir J. (2016) 48:1487–9. 10.1183/13993003.01232-2016 27799392

[11] Ilic M Ilic I. Gender disparities in mortality from infectious diseases in Serbia, 1991-2014: a time of civil wars and global crisis. Epidemiol Infect. (2016) 144:2473–84. 10.1017/S0950268816001345 27483375 PMC9150476

[12] Callahan D. What is a reasonable demand on health care resources? Designing a basic package of benefits. J Contemp Health Law Policy. (1992) 8:1–12. 10118982

[13] Wu Y. China's health care sector in transition: resources, demand and reforms. Health Policy. (1997) 39:137–52. 10.1016/S0168-8510(96)00869-X 10165043

[14] Pagaiya N Phanthunane P Bamrung A Noree T Kongweerakul K. Forecasting imbalances of human resources for health in the Thailand health service system: application of a health demand method. Hum Resour Health. (2019) 17:4. 10.1186/s12960-018-0336-2 30621716 PMC6325808

[15] Blundell R Windmeijer F. Identifying demand for health resources using waiting times information. Health Econ. (2000) 9:465–74. 10.1002/1099-1050(200009)9:6<465::aid-hec525>3.0.co;2-h 10983001

[16] Zhou M Oakes AH Bridges JFP Padula WV Segal JB. Regional supply of medical resources and systemic overuse of health care among medicare beneficiaries. J Gen Intern Med. (2018) 33:2127–31. 10.1007/s11606-018-4638-9 30229364 PMC6258607

[17] Basu K Pak M. Will the needs-based planning of health human resources currently undertaken in several countries lead to excess supply and inefficiency? Health Econ. (2016) 25:101–10. 10.1002/hec.3125 25413332

[18] Wu J Davis-Ajami ML Kevin Lu Z. Impact of depression on health and medical care utilization and expenses in US adults with migraine: a retrospective cross sectional study. Headache. (2016) 56:1147–60. 10.1111/head.12871 27350407

[19] Davis-Ajami ML Lu ZK Wu J. Multiple chronic conditions and associated health care expenses in US adults with cancer: a 2010-2015 medical expenditure panel survey study. BMC Health Serv Res. (2019) 19:981. 10.1186/s12913-019-4827-1 31856797 PMC6924021

[20] Liu X Zhang Q Xu Y Wu X Wang X. Trend analysis of medical expenses in Shenzhen after China's new health-care reforms. Int J Health Plann Manage. (2020) 35:760–72. 10.1002/hpm.2951 31802556

[21] Tobe M Stickley A del Rosario RB Jr. Shibuya K. Out-of-pocket medical expenses for inpatient care among beneficiaries of the National Health Insurance Program in the Philippines. Health Policy Plan. (2013) 28:536–48. 10.1093/heapol/czs092 23048125

[22] Hioki A. Relationship of health services to medical expenses for the national health insurance and certification rate for long-term care insurance services in municipalities. J Epidemiol. (2002) 12:136–42. 10.2188/jea.12.136 12033524 PMC10468335

[23] Coetzee BJ Kagee A. Structural barriers to adhering to health behaviours in the context of the COVID-19 crisis: considerations for low- and middle-income countries. Glob Public Health. (2020) 15:1093–102. 10.1080/17441692.2020.1779331 32524893

[24] Das A. The asthma crisis in low-income communities of color: using the law as a tool for promoting public health. Rev Law Soc Change. (2007) 31:273–314. 17450669

[25] De Pelichy GE Verloo H Gasser J Monod S. Interests and need for strengthening home care in the context of psychological crisis situations—the result of a strong public health policy. Rev Med Suisse. (2020) 16:1741–4. 32969609

[26] Mukherjee D Saxon V. “Psychological Boarding” and community-based behavioral health crisis stabilization. Community Ment Health J. (2019) 55:375–84. 10.1007/s10597-018-0237-9 29380094

[27] Yuen KF Wang X Ma F Li KX. The psychological causes of panic buying following a health crisis. Int J Environ Res Public Health. (2020) 17:3513. 10.3390/ijerph17103513 32443427 PMC7277661

[28] Mukhtar S. Mental health and psychosocial aspects of coronavirus outbreak in Pakistan: psychological intervention for public mental health crisis. Asian J Psychiatr. (2020) 51:102069. 10.1016/j.ajp.2020.102069 32344331 PMC7161472

[29] Pan SL Cui M Qian J. Information resource orchestration during the COVID-19 pandemic: a study of community lockdowns in China. Int J Inf Manage. (2020) 54:102143. 10.1016/j.ijinfomgt.2020.102143 32394997 PMC7211621

[30] Xinhua. The Central Guidance Group Went to Hubei to Inspect the Huoshenshan Hospital‘(2020). Available online at: http://www.gov.cn/guowuyuan/2020-02/03/content_5474082.htm (accessed February 3, 2020).

[31] Council S. Mobilize All Forces to Improve the Production and Supply Capacity of Key Medical Materials (2020). Available online at: http://www.scio.gov.cn/xwfbh/xwbfbh/wqfbh/42311/43082/zy43086/Document/1680729/1680729.htm (accessed May 20, 2020).

[32] Li Q Wei J Jiang F Zhou G Jiang R Chen M. Equity and efficiency of health care resource allocation in Jiangsu Province, China. Int J Equity Health. (2020) 19:211. 10.1186/s12939-020-01320-2 33246458 PMC7694921

[33] Li D Chao J Kong J Cao G Lv M Zhang M. The efficiency analysis and spatial implications of health information technology: a regional exploratory study in China. Health Informatics J. (2020) 26:1700–13. 10.1177/1460458219889794 31793803

[34] Luo Y Yao L Hu L Zhou L Yuan F Zhong X. Urban and rural disparities of personal health behaviors and the influencing factors during the COVID-19 outbreak in China: based on an extended IMB model. Disaster Med Public Health Prep. (2020) 14:1–5. 10.1017/dmp.2020.457 33208220 PMC7985648

[35] Xia Y Li S Meng K. The impact of the COVID-19 on the insurance industry in China. Front Econ Manag. (2020) 1:28. 10.3390/ijerph17165766

[36] Daily E. New Coronary Pneumonia Treatment Costs and Medical Insurance are Reimbursed in Accordance with Regulations (2020). Available online at: http://www.gov.cn/xinwen/2020-04/12/content_5501508.htm (accessed April 12, 2020).

[37] Taxation S. Guidelines on Preferential Tax Policies for Response to the New Coronary Pneumonia Epidemic (2020). Available online at: http://www.gov.cn/xinwen/2020-03/10/content_5489528.htm (accessed March 10, 2020).

[38] Ma C Zhang Y Li Y Wang Y Jiang Y Wang X. Healthcare, insurance, and medical expenditure of the floating population in Beijing, China. Front Public Health. (2020) 8:375. 10.3389/fpubh.2020.00375 32850597 PMC7423999

[39] Sun JY. Welfare consequences of access to health insurance for rural households: evidence from the New Cooperative Medical Scheme in China. Health Econ. (2020) 29:337–52. 10.1002/hec.3985 31814170

[40] Xinhua. Internet Hospital's New Coronary Pneumonia Diagnosis and Treatment Service Platform (2020). Available online at: http://www.gov.cn/xinwen/2020-02/14/content_5478820.htm (accessed February 14, 2020).

[41] He D Gu Y Shi Y Wang M Lou Z Jin C. COVID-19 in China: the role and activities of Internet-based healthcare platforms. Glob Health Med. (2020) 2:89–95. 10.35772/ghm.2020.01017 33330783 PMC7731100

[42] Hu G Li P Yuan C Tao C Wen H Liu Q. Information disclosure during the COVID-19 epidemic in China: city-level observational study. J Med Internet Res. (2020) 22:e19572. 10.2196/19572 32790640 PMC7473703

[43] Xu T Sattar U. Conceptualizing COVID-19 and public panic with the moderating role of media use and uncertainty in China: an empirical framework. Healthcare (Basel). (2020) 8:249. 10.3390/healthcare8030249 32748886 PMC7551926

